# Hydatidiform Mole with Coexisting Normal Pregnancy: A Systematic Review and Individual Participant Data Meta-Analysis

**DOI:** 10.3390/medicina61101781

**Published:** 2025-10-01

**Authors:** Pier Carlo Zorzato, Alberta Ricci, Mariachiara Bosco, Liliana Galli, Laura Luka, Irene Porcari, Rosa Maria Laterza, Veronica Parolin, Michele Milella, Antonio Simone Laganà, Benjamim Ficial, Chiara Casprini, Anna Festi, Stefano Uccella, Simone Garzon

**Affiliations:** 1 Unit of Obstetrics and Gynecology, Department of Surgery, Dentistry, Pediatrics, and Gynecology, Azienda Ospedaliera Universitaria Integrata Verona, University of Verona, Piazzale A. Stefani 1, 37125 Verona, Italy; piercarlo.zorzato@univr.it (P.C.Z.); alberta.ricci@aovr.veneto.it (A.R.); boscomariachiara@gmail.com (M.B.); liliana.galli10@gmail.com (L.G.); lauraluka@gmail.com (L.L.); irene.porcari@aovr.veneto.it (I.P.); chiara.casprini96@gmail.com (C.C.); anna.festi@aovr.veneto.it (A.F.); simone.garzon@univr.it (S.G.); 2 Division of General Gynecology and Gynecologic Oncology, Department of Obstetrics and Gynecology, Medical University of Vienna, 1090 Vienna, Austria; rosa.laterza@meduniwien.ac.at; 3 Karl Landsteiner Society for Special Gynecology and Obstetrics, 1090 Vienna, Austria; 4 Section of Innovation Biomedicine-Oncology Area, Department of Engineering for Innovation Medicine (DIMI), Azienda Ospedaliera Universitaria Integrata Verona, University of Verona, 37125 Verona, Italy; veronica.parolin@aovr.veneto.it (V.P.); michele.milella@univr.it (M.M.); 5 Unit of Obstetrics and Gynecology, “Paolo Giaccone” Hospital, Department of Health Promotion, Mother and Child Care, Internal Medicine and Medical Specialties (PROMISE), University of Palermo, 90127 Palermo, Italy; antoniosimone.lagana@unipa.it; 6 Neonatal Intensive Care Unit, Azienda Ospedaliera Universitaria Integrata Verona, 37125 Verona, Italy; benjamim.ficial@univr.it

**Keywords:** twin pregnancy, complete mole, hydatidiform pregnancy, delivery mode, obstetric management

## Abstract

*Background and Objectives*: This study aimed to evaluate obstetric, neonatal, and oncologic outcomes of pregnancies complicated by a hydatidiform mole coexisting with a live fetus (HMCF) carried beyond viability, and to assess the impact of delivery mode on outcomes. *Materials and Methods*: A systematic review and individual participant data meta-analysis included HMCF cases progressing beyond 23 weeks. Obstetric and neonatal outcomes, delivery patterns, and oncologic risks were analyzed. *Results*: Among 118 pregnancies complicated by HMFC (124 newborns), most were complete moles (87%). Median delivery occurred at 31.6 weeks, with over half before 32 weeks. Common complications included vaginal bleeding (59%), preeclampsia (30%), and hyperthyroidism (18%). Cesarean delivery was performed in 79% of cases, often for mole-related factors, but was not associated with reduced maternal or oncologic risk. Neonatal deaths occurred exclusively in infants delivered ≤32 weeks, highlighting extreme prematurity as the key determinant of survival. Severe preeclampsia was strongly linked to earlier delivery. *Conclusions*: With close monitoring, continuation of HMCF pregnancies is possible. Neonatal mortality is mainly driven by prematurity, which appears to be an indirect consequence of HMFC through the development of mola-associated complications. Cesarean section does not appear to improve maternal and oncologic outcomes. Vaginal delivery can be considered when no standard contraindications exist.

## 1. Introduction

Pregnancies involving a hydatidiform mole coexisting with a live fetus (HMCF)—whether complete (CHMCF) or partial (PHMCF)—are extremely rare, with estimated incidence rates ranging from 1 in 22,000 to 1 in 100,000 pregnancies [1]. CHMCF typically arises in twin gestations, where a complete mole—characterized by hydropic villi, trophoblastic proliferation, and absence of embryonic tissue—coexists with a morphologically and genetically normal fetus supported by a separate healthy placenta. In contrast, PHMCF involves a partial mole with triploid placental tissue and characteristic histopathological changes, usually accompanied by a nonviable or triploid fetus [2].

These pregnancies pose a major clinical dilemma. On the one hand, maternal risks such as severe preeclampsia, hyperthyroidism, and progression to gestational trophoblastic neoplasia (GTN), along with fetal risks including miscarriage, intrauterine demise, and preterm birth, have historically led to pregnancy termination choices upon diagnosis [3,4,5]. On the other hand, more recent evidence indicates that continuation may result in live birth in 20–71% of cases [4,6] without significantly increasing the risk of post-molar GTN compared with termination [7]. This growing recognition has led more patients and clinicians to consider carrying such pregnancies beyond viability.

However, evidence remains scarce regarding optimal obstetric management—particularly the timing and mode of delivery—and how these decisions affect maternal, neonatal, and oncologic outcomes. To address these uncertainties, we performed a systematic review and individual patient data meta-analysis of all reported HMCF cases reaching viability. Our aim is to provide comprehensive outcome data to guide management, inform clinical counseling, and support multidisciplinary decision-making in this rare but challenging condition.

## 2. Materials and Methods

### 2.1. Study Design

This systematic review and meta-analysis was planned before the online search, outlining the study population, definitions, outcome measures, eligibility criteria, and statistical analyses, including subgroup analyses. This study was exempt from Institutional Review Board approval. The methodology followed the Preferred Reporting Items for Systematic Reviews and Meta-Analyses (PRISMA 2020) guidelines [8]. The protocol was registered on PROSPERO (CRD420251036039).

### 2.2. Search Strategy, Eligibility Criteria, and Study Selection

A comprehensive literature search was performed across EMBASE, Scopus, PubMed, Web of Science, and the Cochrane Library from database inception to May 2025. The search was conducted with the support of a certified medical librarian from Biblioteca Meneghetti at the University of Verona. Both MeSH terms and free-text keywords were used to ensure broad coverage, including non-indexed recent publications, and to enable reproducibility across databases. The search strategy was the following: (“Pregnancy, Twin” OR “Pregnancy, Multiple” OR “multiple-pregnancy” OR “multiple-gestation” OR “twin-pregnancy” OR “twin-gestation” OR “twinning-rate” OR “multiple-rate”) AND (“hydatiform-mole” OR “hydatid-mole” OR “hydatidiform-mole” OR “mola-hydatidosa” OR “molar-pregnant”). The search was restricted to human studies, with no language limitations; non-English articles were translated. Reference lists of selected articles were also manually screened to identify additional eligible studies.

Inclusion and exclusion criteria were established a priori by all contributors, and the study question was structured according to the PCC—Population–Concept–Context—framework [9]. Due to the descriptive and exploratory nature of a meta-analysis based on case reports, we adopted the PCC (Population–Concept–Context) framework, as recommended by the Joanna Briggs Institute for structuring reviews of heterogeneous observational data [10,11]. The population included pregnant women with a histologically confirmed diagnosis of complete or partial hydatidiform mole coexisting with at least one live fetus, who delivered at or beyond 23 weeks of gestation. The threshold of 23 weeks was chosen because survival before 23 weeks is rare and almost always associated with significant morbidity, with resuscitation before that gestation age highly debated even in advanced medical contexts [12]. Concept refers to the clinical course, with particular attention to obstetric complications attributable to molar pathology (such as preeclampsia, hyperthyroidism, or abnormal vaginal bleeding), the development of post-molar gestational trophoblastic neoplasia and its management, as well as the gestational age at and mode of delivery, along with fetal outcome and associated factors. Context consists of case reports and case series, often from diverse settings, with varied completeness.

Two reviewers (LG, LL) independently screened the titles and abstracts of articles identified during the initial literature search. The other two reviewers (AR, PCZ) retrieved and independently assessed the full texts of potentially eligible studies. Any disagreements were solved by reexamining the article with an additional reviewer (SG). Only studies with histopathologic confirmation of the molar subtype (complete or partial) and reporting at least one relevant obstetric, fetal, or oncologic outcome were included. Studies were excluded if they reported only pregnancies ending before viability, lacked definitive histologic diagnosis, grouped complete and partial moles without distinction, or were narrative reviews, editorials, or other meta-analyses.

### 2.3. Data Extraction

We developed a standardized form to extract data from the included studies, recording publication details (first author, year, journal, and study design), maternal and neonatal demographics (maternal age, parity, gestational age at diagnosis), clinical presentation, obstetric and molar complications, pregnancy outcomes, mode of delivery, cesarean indications, and oncologic outcomes.

To ensure consistency across reports, clinical definitions and categorizations were standardized, particularly for cesarean indications. We introduced the term “Medical Molar Concerns” to describe cesarean deliveries performed at the physician’s discretion in the absence of conventional obstetric indications, reflecting a precautionary approach to mitigate potential risks of vaginal delivery in the setting of molar pathology (e.g., concerns about hemorrhage, labor obstruction, or other complications).

For analysis, cesarean indications were grouped into two categories: non-mole-related indications—conventional obstetric reasons such as fetal distress, failed induction, or placenta previa, provided these were unrelated to molar disease; mole-related indications—conditions directly attributable to molar pathology, including preeclampsia, hyperthyroidism, abnormal vaginal bleeding, or cases categorized as “medical preference”. Unless otherwise specified, these were considered mole-related complications influencing the decision for surgical delivery (e.g., preeclampsia as an indication for cesarean section was presumed to have developed as a direct complication of the molar gestation and constituted the principal medical reason for performing the cesarean section) [3,5].

### 2.4. Assessment of Risk of Bias

Risk of bias was independently assessed by two reviewers (LG and LL). The Joanna Briggs Institute (JBI) Critical Appraisal Checklist for Case Reports was applied to case reports, while the JBI Checklist for Case Series was used for case series [13]. According to the JBI recommendations, a response of “yes” to each item indicated low risk of bias, whereas any “no” response negatively affected the overall quality of this study. Items marked as “unclear” reflected an indeterminate or unknown risk of bias. Any reviewer discrepancies were resolved through discussion with a third reviewer (SG).

### 2.5. Outcomes and Statistical Analysis

We selected gestational age at delivery and the occurrence of post-molar gestational trophoblastic neoplasia (PTD) as the primary outcomes due to their clinical relevance [12]. Gestational age was prioritized because it is the strongest determinant of neonatal survival. Prematurity also reflects disease severity, as iatrogenic preterm birth typically arises from medical decisions prompted by serious maternal or fetal complications. PTD was chosen as the key cancer-related outcome, given its long-term impact on maternal health [7,14]. Together, these outcomes capture both the immediate obstetric consequences and the long-term oncologic risks of molar pregnancies with a live fetus, providing a comprehensive assessment of maternal and fetal prognosis.

Because our dataset was derived from published case reports—many of which contained detailed individual-level information—we adopted an individual participant data meta-analysis framework [15].

This approach enabled the reanalysis of patient data, allowing for the uniform application of inclusion criteria, harmonization of outcome definitions, and consistent modeling strategies across studies. Baseline maternal and fetal characteristics, as well as clinical outcomes, were summarized using appropriate descriptive statistics. Continuous variables were reported as medians with interquartile ranges, while categorical variables were expressed as absolute frequencies and percentages. We planned to investigate factors associated with gestational age at delivery and post-molar gestational trophoblastic neoplasia (PTD). To investigate the association between possible independent variables and gestational age at delivery, a group comparison stratified by gestational age was planned and performed using the Wilcoxon rank-sum test for non-parametric continuous variables and Fisher’s exact test for categorical variables, as appropriate, to account for small sample sizes.

However, definitive investigation employed multivariable linear mixed-effect regression models. These models included a random intercept for each study to account for clustering of patients within reports and to adjust for inter-study variability. Similarly, to explore predictors of post-molar gestational trophoblastic neoplasia (PTD), a multivariable logistic regression mixed-effect regression model was fitted. Variables included in the multivariable regression models were selected based on clinical judgment and results from univariate analysis when available. The results were presented with 95% confidence intervals (CI) and associated *p*-values. All statistical tests were two-sided, with significance set at *p* < 0.05. Analyses were conducted using R statistical software (v4.1.2; R Core Team 2024), ensuring transparency and reproducibility.

## 3. Results

**Figure 1:**
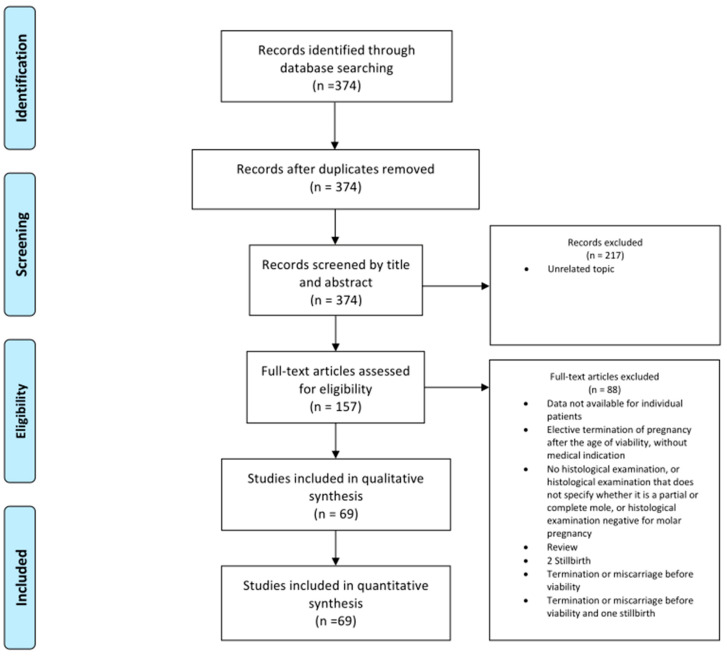
PRISMA flow diagram of reference selection [8].

A total of 374 references were identified through the systematic literature search. After screening, 158 articles were selected for full-text review. Of these, two reports were excluded because they lacked individual patient data; one was excluded for reporting an elective termination of pregnancy beyond the threshold of viability without a medical indication; fifteen were excluded due to the absence of histological confirmation, unclear classification of the molar subtype, or negative findings for molar pregnancy; two were excluded as review articles; and sixty-nine were excluded because the pregnancies ended in miscarriage, stillbirth, or termination before fetal viability. The PRISMA Flowchart illustrates the study selection process (Figure 1).

Ultimately, 69 studies met the inclusion criteria and were included in the final review [2,16,17,18,19,20,21,22,23,24,25,26,27,28,29,30,31,32,33,34,35,36,37,38,39,40,41,42,43,44,45,46,47,48,49,50,51,52,53,54,55,56,57,58,59,60,61,62,63,64,65,66,67,68,69,70,71,72,73,74,75,76,77,78,79,80,81,82,83]. Detailed study characteristics are summarized in Supplementary Materials File S1. Of these, 56 focused on complete hydatidiform mole (CHMCF) [2,16,17,18,20,22,23,24,25,26,27,28,29,30,31,32,33,34,35,36,37,38,39,41,42,43,46,47,48,49,50,54,55,56,57,58,59,60,61,63,64,65,66,67,68,69,70,71,72,73,74,75,76,77,80,81], 12 on partial hydatidiform mole (PHMCF) [19,21,40,44,45,52,53,62,78,79,82,83], and 1 study included both CHMCF and PHMCF cases [38]. The included studies comprised 61 case reports and 8 case series, totaling 117 pregnancies complicated by HMCF for 123 fetuses. In addition, we included one case from our institution that met all the predefined inclusion criteria—a pregnancy with a coexisting partial hydatidiform mole and a viable fetus, described in detail in Supplementary Materials File S2.

### 3.1. Risk of Bias and Quality Assessments

In total, 31 out of 61 case reports were at low risk of bias [16,19,20,21,23,24,25,26,27,29,32,33,35,36,37,38,42,44,50,52,53,55,56,57,70,72,77,78,79,80,83], 27 were at moderate risk [17,18,22,28,30,31,34,39,43,44,45,47,48,54,59,60,62,63,64,65,66,67,68,69,71,73,74,81], and 3 were at high risk [40,58,82]. Case reports with a low risk of bias consistently provided comprehensive reporting of patient clinical details, including diagnostic workup, mode of delivery, postpartum course, and any complications. Conversely, those with moderate risk often lacked data, diagnostic procedures, and descriptions of post-intervention outcomes; however, they generally provided thorough narratives of clinical presentation, mode of delivery, adverse events, and key lessons learned. Reports assessed as having a high risk of bias usually missed critical clinical information, such as patient demographics, initial presentation, diagnostic assessments, postpartum course, and details regarding complications and their management. These omissions significantly hinder the interpretability, generalizability, and methodological integrity of these reports.

Two out of eight case series were at low risk of bias [41,51], and six were at moderate risk [2,46,49,61,75,76]. Low-risk case series featured well-defined inclusion criteria, suitable methods for measuring and diagnosing conditions, consecutive patient inclusion, and detailed medical history documentation.

However, some studies lacked adequate reporting on patient demographics, follow-up procedures, and statistical analysis. Case series assessed as having a moderate risk of bias generally failed to provide sufficient detail regarding patient demographic characteristics, clinical presentation upon hospital admission, follow-up protocols, and statistical methods. Bias assessment is summarized in Supplementary Materials File S3—Reported cases and their risk of bias according to the Joanna Briggs Institute (JBI) Critical Appraisal Checklist for Case Reports and in Supplementary Materials File S4—Reported cases and their risk of bias according to the Joanna Briggs Institute (JBI) Critical Appraisal Checklist for Case Series).

### 3.2. Population Characteristics and Outcomes

We identified 118 HMFC pregnancies that were carried beyond 23 weeks of gestation, including 113 singleton pregnancies, four twin pregnancies, and one triplet pregnancy, for a total of 124 live births (Table 1).

The majority involved complete moles (102 cases, 87%), while partial moles comprised 16 cases (13%). The median maternal age was 30.0 years (IQR, 27.0–34.4), with most deliveries occurring in nulliparous women (59%; 38/63). Conception was facilitated via assisted reproductive technologies (ART) in 50% of cases (29/57), including ovulation induction, in vitro fertilization, and intracytoplasmic sperm injection (ICSI). Maternal complications during pregnancy were preeclampsia, which occurred in 30% cases (24/79), hyperthyroidism (18%; 14/79), and vaginal bleeding (59%; 47/79).

The median gestational age at delivery was 31.6 weeks (IQR, 27.9–35.7), with 55% deliveries occurring before 32 weeks of gestation. Neonatal data showed a median birth weight of 1567 g (IQR, 1003–2263). Among neonates, 41 (55%) were female and 34 (45%) were male. Cesarean section was the most common mode of delivery, accounting for 78% of cases. Among cases with a documented indication for cesarean delivery, 44% (27/61) were “Cesarean Section for Mole-Related Indications.” In contrast, the remaining 56% (34/61) of cesarean sections were performed for obstetric reasons unrelated to molar disease and labeled as “Cesarean Section for Non-Mole-Related Indications.”

An in-depth analysis of indications for cesarean section revealed that the most frequently reported reason was “Medical Molar Concerns” (21.7%). For instance, Giorgione et al. reported three cesarean sections for unspecified “preterm labor [2],” while Hamanoue et al. described a prophylactic cesarean in a patient with preterm labor, considering the increased risk associated with a complete mole [30]. Al Mouallem et al. performed a cesarean at 38 weeks without documenting clear maternal or fetal indications [48]. Similarly, Wang et al. scheduled an elective cesarean at 36–37 weeks in a high-risk molar pregnancy following corticosteroid administration [16]. Yayna et al. cited the presence of a viable fetus in a molar pregnancy as the reason for cesarean in a case of preterm labor [36]. Rao et al. and Mora-Palazuelos et al. also described cesareans for preterm labor [56,79]; in the latter case, the procedure was motivated by concerns about uterine atony and possible hysterectomy, which was ultimately avoided. Lin et al. performed a term cesarean due to worries over potential trophoblastic metastasis triggered by uterine contractions [83]. Wax et al. delivered 36 weeks after corticosteroid administration in preparation for a planned hysterectomy [58]. Zeng et al. reported a cesarean at 29 weeks following hospitalization for contractions [52], and Chu et al. performed a cesarean at 24 + 2 weeks due to preterm labor associated with maternal fever [82].

In addition to medical preference, other common indications for cesarean section included vaginal bleeding (20.0%) and preeclampsia (18.3%). Less frequent but relevant reasons included breech presentation (11.7%), abnormal cardiotocography (11.7%), and placenta previa (6.7%). Rare indications involved previous cesarean section (3.3%), reduced fetal movements (1.7%), intrauterine growth restriction (1.7%), and suspected placenta accreta spectrum (1.7%).

Regarding post-molar outcomes, gestational trophoblastic neoplasia (PTD) occurred in 17 of 74 cases (23%), chemotherapy was administered in 19 of 74 (26%), and metastatic disease developed in 6 of 74 (8.5%). The mean time to β-hCG normalization was 95 days (SD 81).

### 3.3. Factors Associated with Gestational Age at Delivery

When comparing patients who delivered after 32 weeks of gestation (n = 39) with those who delivered at or before 32 weeks (n = 48), no statistically significant differences were found in baseline maternal characteristics. Maternal complications such as preeclampsia (29% vs. 30%, *p* > 0.999), hyperthyroidism (14% vs. 19%, *p* = 0.763), and vaginal bleeding (51% vs. 65%, *p* = 0.253) did not differ significantly between the two groups. Neonatal birthweight was significantly lower in the ≤32-week group (median 1018 g; IQR 700–1272) than in the >32-week group (median 2310 g; IQR 2015–2705) (*p* < 0.001). The perinatal mortality rate was 11.2% for the entire cohort, with 14 documented neonatal deaths all before 32 weeks (Table 2).

The overall cesarean delivery rate did not differ significantly between age groups (74% vs. 81%, *p* = 0.449). Cesarean sections for mole-related indications were performed in 50% of cases in the >32-week group and 39% in the ≤32-week group (*p* = 0.435). Indications for cesarean did not show significant differences between groups (*p* = 0.191). The cesareans attributed to “Medical Molar Concerns” as defined in the “Methods” paragraph were more common in the >32-week group (33% vs. 14%).

A linear mixed-effect model was employed to assess the impact of mole-related maternal complications on the timing of delivery. Covariates included preeclampsia, hyperthyroidism, vaginal bleeding, and mole classification (complete vs. partial). The analysis revealed that none of the examined mole-related maternal complications were significantly associated with gestational age at delivery. Preeclampsia was associated with a non-significant reduction in gestational age of 0.82 weeks (β = −0.82; 95% CI: −3.1 to 1.5; *p* = 0.48). Similarly, hyperthyroidism showed a non-significant association (β = −0.72; 95% CI: −3.4 to 2.0; *p* = 0.61), as did vaginal bleeding (β = −0.44; 95% CI: −2.5 to 1.6; *p* = 0.68). When comparing molar subtypes, pregnancies with partial moles showed a non-significant reduction in gestational age compared to those with complete moles (β = −0.58; 95% CI: −3.3 to 2.1; *p* = 0.67).

To further investigate whether mole-related maternal complications were linked to earlier delivery, we examined them as the indication for cesarean section. Cases with “Cesarean Section for Mole-Related Indications” included reasons like excessive uterine size, high β-hCG levels, and obstetric complications associated with mola—such as preeclampsia, hyperthyroidism, vaginal bleeding, or trophoblastic invasion. It also covered cases labeled as “Medical Molar Concerns,” where a cesarean was chosen prophylactically or out of caution without clear obstetric reasons, due to perceived risks from molar tissue. When analyzed as a single group, cesarean sections performed for these mole-related indications were associated with a non-significant reduction in gestational age at delivery of 1.8 weeks (β = −1.8; 95% CI: −4.1 to 0.44; *p* = 0.11). When analyzed separately, “Cesarean Section for Mole-Related Indications,” in which the indication was preeclampsia, was associated with a statistically significant reduction in gestational age at delivery (β = –2.9 weeks; 95% CI: −5.8 to −0.05; *p* = 0.046). “Cesarean Section for Mole-Related Indications,” in which the indication was vaginal bleeding, was not associated with a significant difference in gestational age at delivery (β = 0.02; 95% CI: −2.9 to 2.9; *p* > 0.99).

### 3.4. Comparison of Oncological Outcomes According to Mode of Delivery

The incidence of PTD was similar between those who delivered by cesarean section (n = 69; 24%) and those who delivered vaginally (n = 18; 20%; *p* > 0.999), as were the rates of chemotherapy administration (27% vs. 20%, *p* = 0.746) and metastases development (8.8% vs. 6.7%, *p* > 0.999). The median time to β-hCG normalization was shorter in the vaginal delivery group (53 days, IQR 30–90) compared to the cesarean section group (70 days, IQR 51–117). However, this difference was not statistically significant (*p* = 0.345). At the multivariable multilevel logistic regression analysis, cesarean delivery was not significantly associated with an increased risk of PTD (OR 1.31; 95% CI 0.29–5.79; *p* = 0.73), as well as gestational age at delivery (OR 1.01; 95% CI 0.89–1.14; *p* = 0.91). Compared to a complete mole, a partial mole was associated with lower odds of PTD, although this reduction was not statistically significant (OR 0.44; 95% CI 0.09–2.22; *p* = 0.32).

## 4. Discussion

This study provides the most comprehensive review of pregnancies with a hydatidiform mole concomitant to a live fetus (HMCF), a rare and high-risk condition. Among 118 pregnancies complicated by HMFC and 124 newborns beyond 23 weeks from 69 studies, four key findings emerged: (1) Prematurity was strikingly frequent—over half of all deliveries occurred before 32 weeks, and all neonatal deaths were confined to this group, underscoring extreme prematurity as the main determinant of adverse neonatal outcomes. (2) Preeclampsia was observed in approximately one-third of cases. While our multivariate analysis did not identify preeclampsia alone as a predictor of delivery timing, cesarean delivery for preeclampsia (used as a proxy for severe disease) was associated with significantly earlier delivery, suggesting that severe preeclampsia may be a major driver of prematurity. (3) Cesarean delivery was the predominant mode of birth (79%), far exceeding general obstetric averages. Nearly half of these procedures were performed for mole-related reasons—including preeclampsia, vaginal bleeding, hyperthyroidism, or physician-initiated “Medical Molar Concerns”—rather than traditional obstetric indications. The prevalence of “Medical Molar Concerns” reflects uncertainty in managing HMCF and highlights the absence of evidence-based guidelines. Importantly, cesarean section did not appear to improve obstetric or oncologic outcomes. (4) The incidence of postmolar gestational trophoblastic disease (PTD/GTN) was 26–35%, higher than in singleton molar pregnancies, but unrelated to gestational age at delivery or mode of birth. This suggests that oncologic risk is primarily driven by the biological behavior of the molar tissue rather than obstetric management.

In our study, the median gestational age at delivery was 31.6 weeks (IQR 27.9–35.7), consistent with prior reports. Multiparity was observed in 41% of cases, supporting evidence that parity is not a major risk factor for molar disease. In contrast, the median maternal age of 30.0 years (IQR 27.0–34.5) does not align with traditionally high-risk groups (<20 or >40 years) [84]. Assisted reproductive technologies (ART) were used in 50% of pregnancies, suggesting a possible association with molar pregnancy, though existing literature generally reports no increased risk compared with spontaneous conception [85,86]. Our study design does not allow for causal inference [85,86]. Maternal complications were frequent and comparable to published trends: preeclampsia occurred in 31%, hyperthyroidism in 18%, and vaginal bleeding in 59%—higher than the 33% usually cited, perhaps reflecting referral or reporting differences [4,5].

Prematurity was striking: 56% of deliveries occurred before 32 weeks and 25% before 28 weeks, compared with ~10% in the general population [87,88]. All neonatal deaths were confined to those born ≤32 weeks, particularly <28 weeks, including one case at 32 weeks with preeclampsia and intrauterine growth restriction [87,88]. One case involved a fetus delivered at 32 weeks to a mother affected by preeclampsia and severe fetal growth restriction [2,25,43,44,51,54,66,68,69,76]. These findings suggest that extreme prematurity is the principal determinant of neonatal mortality.

Preeclampsia may contribute to preterm delivery. While preeclampsia alone did not significantly alter gestational age in our multivariate analysis, severity could not be assessed from available data. As a proxy, we assumed cesarean delivery for preeclampsia reflected severe disease [89]. This aligns with guidelines recommending early delivery for severe preeclampsia [90,91,92]. Indeed, cesarean for preeclampsia was associated with significantly earlier delivery (~2.9 weeks), supporting severe preeclampsia as a major driver of prematurity in HMCF. Therefore, neonatal mortality does not appear to be a direct consequence of HMCF but an indirect consequence through the development of mola-associated complications.

Cesarean delivery occurred in 79% of cases, far above global averages (19–32%) or low-risk pregnancies (<15%) [93]. Indications differed from the general population, where labor arrest, previous cesarean or macrosomia are common [94,95]. Here, nearly half were due to mole-related factors—preeclampsia (18%), vaginal bleeding (20%), hyperthyroidism, or “Medical Molar Concerns” (21%).

Notably, 21% were attributed to “Medical Molar Concerns”—physician-initiated decisions made without an absolute medical condition, often in early labor. This reflects the uncertainty and variability in managing HMCF pregnancies. In many cases, surgical delivery was chosen as a precautionary measure rather than based on documented maternal or fetal compromise. Concerns about complications such as hemorrhage, uterine rupture, or trophoblastic dissemination often drive these decisions. Without standardized guidelines, clinical judgment usually relies on perceived risk rather than evidence-based criteria, likely contributing to the overestimated cesarean section rate observed in this population. Our results suggest that the mode of delivery is not associated with obstetric outcomes. Therefore, available evidence does not support an elective cesarean section as a preventive measure for the safety of the mother and newborn in HMFC, unless otherwise indicated for a concurrent obstetric pathology that in itself has an indication for caesarean section. Notably, a protective effect was not observed even for oncologic outcomes.

The risk of postmolar gestational trophoblastic disease (PTD) [96] remains a major concern. While PTD risk after singleton molar pregnancy is ~2–3% for CHM and <0.5% for PHM, it rises to 26–35% in HMCF [3]. However, our analysis showed no association between PTD risk and either gestational age at delivery or delivery mode. Vaginal birth yielded comparable outcomes, including chemotherapy need, metastasis, and β-hCG normalization. Thus, oncologic outcomes appear driven by molar tissue biology rather than delivery management. Evidence remains insufficient to determine whether earlier interruption (<23 weeks) reduces PTD risk.

Strengths of this review include its systematic methodology and comprehensive capture of all reported cases, providing the most detailed synthesis available for this rare condition. However, important limitations must be acknowledged. Case-based data are inherently heterogeneous, with variable reporting of complications, delivery indications, and oncologic outcomes. Incomplete data limited subgroup analyses, and assumptions and harmonization (e.g., cesarean for preeclampsia as a surrogate for severity) may oversimplify clinical complexity. The exclusion of pregnancies ending before 23 weeks introduces selection bias. By focusing only on viable gestations, the analysis cannot address potential differences in oncologic risk between continued and interrupted pregnancies. Finally, conclusions on the role of cesarean section are limited by the possible risk of biases in classifying indications. In particular, some cases of “Medical Molar Concerns” may be classified differently if more details are provided. Moreover, possible adverse outcomes of vaginal delivery may have been missed due to the high prevalence of cesarean section.

Despite these limitations, our findings highlight the urgent need for standardized definitions, prospective registries, and uniformed outcome reporting. Beyond registries, the establishment of international collaboration networks for rare diseases could facilitate data sharing, improve case ascertainment, and accelerate the development of evidence-based guidelines for HMCF. Only through coordinated global efforts can more robust data be generated to guide clinical decision-making in this challenging condition.

## 5. Conclusions

Our analysis suggests that continuing pregnancy in cases of hydatidiform mole coexisting with a live fetus (HMCF) is feasible and can be safe when supported by close maternal–fetal monitoring. No maternal deaths were reported, and continuation did not increase the risk of postmolar malignant progression. This is an important finding for patient counseling, underscoring that termination is not always necessary and that individualized management is appropriate. Neonatal outcomes were primarily determined by gestational age at delivery, with extreme prematurity emerging as the leading cause of neonatal mortality. Although the factors driving early delivery remain uncertain, severe preeclampsia—likely consequence of HMFC—appears to play a major role. Extending pregnancy beyond the threshold of viability should therefore be prioritized whenever maternal and fetal conditions remain stable. The cesarean delivery rate was strikingly high (79%), with a proportion reflecting precautionary decisions rather than standard obstetric indications. Importantly, we found no evidence that cesarean section reduces the risk of gestational trophoblastic neoplasia (GTN) or improves oncologic outcomes. Vaginal delivery should thus be considered a safe and valid option when obstetric contraindications are absent, while cesarean section should be reserved for conventional indications—related or not related to HMFC—rather than performed preemptively for molar pathology. Given the rarity and complexity of HMCF, we recommend management in specialized centers with multidisciplinary expertise in high-risk obstetrics and gestational trophoblastic disease, institutional readiness for complications such as severe preeclampsia and hemorrhage, standardized classification of cesarean indications to avoid unnecessary intervention, and the establishment of international registries to strengthen prospective data and guide future evidence-based recommendations.

## Figures and Tables

**Table 1:** Characteristics of HMCF Pregnancies.

Summary Characteristics of HMCF Pregnancies		
	Total number of cases	Missing
Pregnancies complicated by HMFC	118	
Newborns beyond 23 sg (n)	124	
Age (years) ^1^	30.0 (27.0–34.5)	41
Multiparous	26 (41%)	60
ART	29 (50%)	66
Molar type		
– Complete	102 (87%)	
– Partial	16 (13%)	
**Obstetric Outcomes and Delivery Data**		
Preterm delivery <32 w	48 (55%)	37
GA at delivery (weeks) ^1^	31.6 (27.9–35.7)	37
Cesarean delivery	69 (78%)	36
CS Indication		63
– Breech presentation	7 (11%)	
– Suspect PAS	1 (1.6%)	
– IUGR	1 (1.6%)	
– Vaginal bleeding	12 (20%)	
– Chronic kidney failure	1 (1.6%)	
– Preeclampsia	11 (18%)	
– Low fetal movements	1 (1.6%)	
– Placenta previa	4 (6.6%)	
– CTG	7 (11%)	
– Previous CS	2 (3.3%)	
– Medical Molar Concerns	13 (21%)	
– Sepsis	1 (1.6%)	
Cesarean Section for Mole-Related Indications	27 (44%)	63
Cesarean Section for Non-Mole-Related Indications	34 (56%)	63
Birthweight (g) ^1^	1567 (1003–2263)	48
Neonatal sex		49
– Female	41 (55%)	
– Male	34 (45%)	
Preeclampsia	24 (30%)	45
Hyperthyroidism	14 (18%)	45
Vaginal bleeding	47 (59%)	45
**Oncologic Outcomes**		
Postmolar GTN (PTD)	17 (23%)	50
Chemotherapy	19 (26%)	50
Metastasis	6 (8.3%)	52
Time to β-hCG normalization (days) ^2^	95 (81)	78

^1^ Median (IQR); ^2^ Mean (SD); HMCF: hydatidiform mole coexisting with a live fetus; ART: Assisted Reproductive Technologies; CS: Cesarean Section; CTG: Cardiotocography; GA: Gestational Age; IUGR: Intrauterine Growth Restriction; PAS: Placenta Accreta Spectrum; GTN: Gestational Trophoblastic Neoplasia; PTD: Postmolar Trophoblastic Disease; Medical Molar Concerns: defined in the “Methods”; Cesarean Section for Mole-Related Indications: defined in the “Methods”; Cesarean Section for Non-Mole-Related Indications: defined in the “Methods”.

**Table 2:** Comparison of Characteristics HMCF Pregnancies by Gestational Age at Delivery.

	Overall (n = 87)	>32 Weeks (n = 39)	≤32 Weeks (n = 48)	*p*-Value ^2^
Age (years) ^1^	30.0 (27.0–34.8)	30.0 (27.0–36.0)	29.0 (28.0–33.0)	0.390
Multiparous	26 (41%)	9 (32%)	17 (49%)	0.209
Assisted Reproductive Technology	28 (49%)	10 (42%)	18 (55%)	0.424
Molar type				>0.999
– Complete	71 (82%)	32 (82%)	39 (81%)	
– Partial	16 (18%)	7 (18%)	9 (19%)	
Gestational Age at delivery (weeks) ^1^	31.6 (27.9–35.7)	36.0 (34.0–38.0)	27.9 (25.0–29.9)	<0.001
Cesarean delivery (CS)	68 (78%)	29 (74%)	39 (81%)	0.449
CS indication				0.191
– Breech presentation	7 (12%)	4 (17%)	3 (8.3%)	
– Suspect PAS	1 (1.7%)	0 (0%)	1 (2.8%)	
– IUGR	1 (1.7%)	1 (4.2%)	0 (0%)	
– Vaginal bleeding	11 (18%)	3 (13%)	8 (22%)	
– Chronic kidney failure	1 (1.7%)	0 (0%)	1 (2.8%)	
– Preeclampsia	11 (18%)	4 (17%)	7 (19%)	
– Low fetal movements	1 (1.7%)	0 (0%)	1 (2.8%)	
– Placenta previa	4 (6.7%)	0 (0%)	4 (11%)	
– Cardiotocography alteration	7 (12%)	2 (8.3%)	5 (14%)	
– Previous CS	2 (3.3%)	2 (8.3%)	0 (0%)	
– Medical Molar Concerns	13 (22%)	8 (33%)	5 (14%)	
– Sepsis	1 (1.7%)	0 (0%)	1 (2.8%)	
Cesarean Section for Mole-Related Indications	26 (43%)	12 (50%)	14 (39%)	0.435
Cesarean Section for Non-Mole-Related Indications	34 (57%)	12 (50%)	22 (61%)	
Neonatal birthweight (g) ^1^	1590 (1009–2265)	2310 (2015–2705)	1018 (700–1272)	<0.001
Neonatal sex				0.159
– Female	41 (55%)	21 (66%)	20 (48%)	
– Male	33 (45%)	11 (34%)	22 (52%)	
Preeclampsia	23 (29%)	10 (29%)	13 (30%)	>0.999
Hyperthyroidism	13 (17%)	5 (14%)	8 (19%)	0.763
Vaginal bleeding	46 (59%)	18 (51%)	28 (65%)	0.253

^1^ Median (IQR); ^2^ Mean (SD);ART: Assisted Reproductive Technologies; CS: Cesarean Section; CTG: Cardiotocography; GA: Gestational Age; IUGR: Intrauterine Growth Restriction; PAS: Placenta Accreta Spectrum; GTN: Gestational Trophoblastic Neoplasia; PTD: Postmolar Trophoblastic Disease; Medical Molar Concerns: defined in the “Methods”; Cesarean Section for Mole-Related Indications: defined in the “Methods”; Cesarean Section for Non-Mole-Related Indications: defined in the “Methods”.

## Supplementary Materials

The following supporting information can be downloaded at: https://www.mdpi.com/article/10.3390/medicina61101781/s1, Supplementary Materials File S1: Studies included in the meta-analysis. Supplementary Materials File S2: Case report of hydatidiform mole coexisting with a live fetus. Supplementary Materials File S3: Reported cases and their risk of bias according to the Joanna Briggs Institute (JBI) Critical Appraisal Checklist for Case Reports. Supplementary Materials File S4: Reported cases and their risk of bias according to the Joanna Briggs Institute (JBI) Critical Appraisal Checklist for Case Series. Supplementary Materials File S5: Bar chart of risk of bias assessment.
