# Supplementary material for: Hydatidiform Mole with Coexisting Normal Pregnancy: A Systematic Review and Individual Participant Data Meta-Analysis
Source: Medicina (Kaunas). 2025 Oct 1;61(10):1781. doi: 10.3390/medicina61101781 (PMC12566089; doi:10.3390/medicina61101781)
Supplement: Supplementary file 1 [file medicina-61-01781-s001.zip › supp-medicina-3822615/Supplementary Materials File S1.pdf]

Supplementary Material 1: Studies included in the meta-analysis.								
**Author and year of publication**	**Study design**	**Type of hydatidiform mole**	**Clinical presentation of molar pregnancy**	**Gestational age at delivery (weeks + days)**	**Mode of delivery**	**Main indication for CS**	**N of live neontes for each pregnant**	**PTD**
**Shazly et al., 2012**	Case report	Complete	Preeclampsia, hyperthyroidism, vaginal bleeding		CS	Vaginal bleeding	1	No
**Chao et al., 1999**	Case report	Complete	Preeclampsia, hyperthyroidism, vaginal bleeding	25	CS	Vaginal bleeding	3	Yes
**Suriet al., 2009**	Case report	Complete	Vaginal bleeding	28	CS	Sepsis	1	Yes
**Ko et al., 2007**	Case report	Complete	Preeclampsia	33	CS	Bigeminal pregnency	2	No
**Van de Geijn et al., 1992**	Case report	Complete	Vaginal bleeding	24+5	VD		2	No
**Johnston et al., 2000**	Case report	Complete	Vaginal bleeding	30	CS	Breech presentation	1	No
**Singh et al., 2011**	Case report	Complete	Preeclampsia and vaginal bleeding	36+6	CS	Preeclampsia	1	No
**Peng et al., 2014**	Case report	Complete	None	37	CS	Breech presentation	1	Yes
**Cheng et al., 1995**	Case report	Complete	Vaginal bleeding	29	CS	Placenta previa	1	No
**Miskovic et al., 2006**	Case report	Complete	None	37	VD		1	No
**Kan et al., 2018**	Case report	Complete	Preeclampsia and vaginal bleeding	35	CS	Breech presentation	1	No
**Rajesh et al., 2000**	Case report	Complete	Vaginal bleeding	24	VD		2	No
**Miller et al., 1993**	Case report	Complete	Vaginal bleeding	38	VD		1	No
**Vaisbuch et al., 2005**	Case report	Complete	Preeclampsia	26	CS	Preeclampsia	1	No
**Dolapcioglu et al., 2009**	Case report	Complete	Vaginal bleeding	29	CS	Vaginal bleeding	1	No
**Lee et al., 2010**	Case report	Complete	Hyperthyroidism and vaginal bleeding	38	VD		1	Yes
**Moini et al., 2011**	Case report	Complete	Vaginal bleeding	39	CS	Previous CS	1	No
**Buke et al., 2014**	Case report	Complete	Vaginal bleeding	32	CS	Placenta previa	1	No
**Montes-de-Oca-Valero et al., 1999**	Case report	Complete	Preeclampsia and vaginal bleeding	27+6	CS	Placenta previa	1	No
**Piura et al., 2008**	Case report	Complete	Vaginal bleeding	28	CS	Breech presentation	1	No
**Bovicelli et al., 2004**	Case report	Complete	Vaginal bleeding	31	CS	Low fetal moovements	1	No
**Wax et al., 2003**	Case report	Complete	No information	36	CS	“Medical Molar Concerns”	1	No
**Abbiet al., 1999**	Case report	Complete	Vaginal bleeding	37	CS	Breech presentation	1	No
**Hamanoue et al., 2006**	Case report	Complete	Vaginal bleeding	33	CS	“Medical Molar Concerns”	1	No
**Klatt et al., 2006**	Case report	Complete	Vaginal bleeding	31	CS	Vaginal bleeding	1	No
**Aguilera et al., 2012**	Case report	Complete	Preeclampsia and vaginal bleeding	34	CS	Vaginal bleeding	1	Yes
**Johnson et al., 2019**	Case report	Complete	Vaginal bleeding	34	CS	Previous CS	1	No
**Raj et al., 2019**	Case report	Complete	Preeclampsia, hyperthyroidism, vaginal bleeding	24	CS	Preeclampsia	1	No
**Alpay et al., 2021**	Case report	Complete	Preeclampsia	26	CS	Preeclampsia	1	Yes

**Lipiet al., 2020**	Case report	Complete	Preeclampsia	33	CS	Preeclampsia	1	Yes
**Albayrak et al., 2010**	Case report	Complete	None	33	CS	Breech presentation	1	No
**Al Mouallem et al., 2022**	Case report	Complete	Vaginal bleeding	38	CS	"Medical Molar Concerns"	1	No
**Jung, 2023**	Case report	Complete	Vaginal bleeding	28+5	CS	Suspected PAS	1	Yes
**McHenry et al., 2021**	Case report	Complete	None	35	CS	Non-reassuring CTG	1	No
**Wang et al., 2023**	Case report	Complete	Vaginal bleeding	36	CS	"Medical Molar Concerns"	1	
**Taira et al., 2021**	Case report	Complete	Preeclampsia	37	VD		1	Yes
**Yayna et al., 2023**	Case report	Complete	Hyperthyroidism	33	CS	Medical preference	1	No
**Bursać et al., 2023**	Case report	Complete	Hyperthyroidism and vaginal bleeding	38	CS	IUGR	1	No
**Tsakiridis et al., 2021**	Case report	Complete	Vaginal bleeding	35	CS	Vaginal bleeding	1	No
**Thompson et al., 2022**	Case report	Complete	Hyperthyroidism and vaginal bleeding	27+6	CS	Vaginal bleeding	1	Yes
**Okumura et al., 2014**	Case report	Complete	Preeclampsia	32	CS	Preeclampsia	1	No
**Albers et al., 2001**	Case report	Complete	None	40	VD		1	No
**Rao et al., 2015**	Case report	Complete	Vaginal bleeding	31	CS	"Medical Molar Concerns"	1	No
**Lambert-Messerlian et al., 2005**	Case report							
		Complete		23	VD		1	No
		Complete		28	CS	Non-reassuring CTG	1	Yes
**Liet al., 2024**	Case report	Complete	Preeclampsia and vaginal bleeding	30+5	CS	Bigeminal pregnancy	2	Yes
**Soriano-Estrella et al., 2024**	Case report	Complete	Vaginal bleeding	25+1	CS	Placenta previa	1	
**Makary et al., 2010**	Case report	Complete	Preeclampsia	25	CS	Preeclampsia	1	Yes
**Freis et al., 2016**	Case report	Complete	Vaginal bleeding	31.4	CS	Vaginal bleeding	1	No
**Shuja and Rauf, 2024**	Case report	Complete	Vaginal bleeding	32	CS	Vaginal bleeding	1	No
**Kawasaki et al., 2016**	Case report	Partial	Preeclampsia	25+3	CS	Breech presentation	1	No
**Zeng et al., 2019**	Case report	Partial	Vaginal bleeding	29+6	CS	"Medical Molar Concerns"	1	No
**Chu et al., 2004**	Case report	Partial	Vaginal bleeding	24+2	CS	"Medical Molar Concerns"	1	No
**Rai et al., 2014**	Case report	Partial	Vaginal bleeding	36	CS	Breech presentation	1	No
**Rathod et al., 2015**	Case report	Partial	Vaginal bleeding	28	VD		1	No
**Tolcha et al., 2022**	Case report	Partial	Preeclampsia and hyperthyroidism	29+6	CS	Breech presentation	1	No
**Libretti et al., 2023**	Case report	Partial	None	38+1	VD		1	No
**Rajasekaran et al., 2021**	Case report	Partial	None	28+2	CS	Non-reassuring CTG	2	No
**Qu et al., 2022**	Case report	Partial	Vaginal bleeding	38+2	CS	Non-reassuring CTG	1	No
**Mora-Palazuelos et al., 2023**	Case report	Partial	None	35+3	CS	"Medical Molar Concerns"	1	
**Lin et al., 2021**	Case report	Partial	None	40+2	CS	"Medical Molar Concerns"	1	Yes
**Copeland and Stanek., 2010**	Case report	Partial	None	28	CS	Worsening maternal CRF	1	No

**Fishman et al., 1998**	Case series							
		Complete		26	VD		1	Yes
		Complete		34	CS	NI	1	Yes
**Niemann et al., 2007**	**Case series**	Complete	Preeclampsia and vaginal bleeding	27+6	CS	Preeclampsia	1	No
**Massardier et al., 2009**	**Case series**							
		Complete	None	25	VD		1	No
		Complete	Preeclampsia	38+2	VD		1	No
		Complete	Hyperthyroidism	27+2	CS	Non-reassuring CTG	1	No
**Kutuk et al., 2014**	**Case series**	Complete	Preeclampsia	34	VD		1	No
**Giorgione et al., 2017**	**Case series**							
		Complete	None	34	CS	"Medical Molar Concerns"	1	Yes
		Complete	None	27	CS	Non-reassuring CTG	1	No
		Complete	Hyperthyroidism	26	CS	"Medical Molar Concerns"	1	No
		Complete	Hyperthyroidism and vaginal bleeding	37	VD		1	No
		Complete	Vaginal bleeding	30	CS	"Medical Molar Concerns"	1	No
		Complete	None	23	VD		1	No
**Lin et al., 2017**	**Case series**							
		Complete					1	
		Complete					1	
		Complete					1	
		Complete					1	
		Complete					1	
		Complete					1	
		Complete					1	
		Complete					1	
		Complete					1	
		Complete					1	
		Complete					1	
		Complete					1	
		Complete					1	
		Complete					1	
		Complete					1	
		Complete					1	
		Complete					1	
		Complete					1	
		Complete					1	
		Complete					1	
		Complete					1	
		Complete					1	
		Complete					1	

		Complete					1	
		Complete					1	
		Complete					1	
		Complete					1	
		Complete					1	
		Complete					1	
		Complete					1	
		Complete					1	
		Complete					1	
		Complete					1	
		Complete					1	
		Complete					1	
		Complete					1	
**Liang et al., 2022**	**Case series**							
		Complete	Preeclampsia and vaginal bleeding	37	CS		1	No
		Complete	None	38	CS		1	No
		Partial	Vaginal bleeding	34	CS		1	Yes
		Partial	Preeclampsia	32	CS		1	No
**Lu et al., 2022**	**Case series**	Complete		34	CS		1	Yes

Abbreviations: CS, cesarean section; VD, vaginal delivery; CTG, cardiotocography; PTD, persistent trophoblastic disease; Empty cells, no information
