# Supplementary material for: Hydatidiform Mole with Coexisting Normal Pregnancy: A Systematic Review and Individual Participant Data Meta-Analysis
Source: Medicina (Kaunas). 2025 Oct 1;61(10):1781. doi: 10.3390/medicina61101781 (PMC12566089; doi:10.3390/medicina61101781)
Supplement: Supplementary file 1 [file medicina-61-01781-s001.zip › supp-medicina-3822615/Supplementary Materials File S3.pdf]

Supplementary Material 3: Reported cases and their risk of bias according to the Joanna Briggs Institute (JBI) Critical Appraisal Checklist for Case Reports										
**Author**	**Year**	**Were Patient's Demographic Characteristics Clearly Described?**	**Was the Patient's History Clearly Described and Presented as a Timeline?**	**Was the Current Clinical Condition of the Patient on Presentation Clearly Described?**	**Were Diagnostic Tests or Assessment Methods and the Results Clearly Described?**	**Was the Intervention(s) or Treatment Procedure(s) Clearly Described?**	**Was the Post-Intervention Clinical Condition Clearly Described?**	**Were Adverse Events (Harms) or Unanticipated Events Identified and Described?**	**Does the Case Report Provide Takeaway Lessons?**	**Risk of bias**
Shazly	2012	yes	yes	yes	yes	yes	yes	yes	yes	Low
Chao	1999	no	yes	yes	yes	yes	yes	yes	yes	Low
Suri	2009	no	yes	yes	yes	yes	no	yes	yes	Moderate
Ko	2007	no	yes	yes	no	yes	no	yes	yes	Moderate
van de Geijn E	1992	no	yes	yes	yes	yes	no	yes	yes	Moderate
Johnston	2000	no	yes	yes	yes	yes	yes	no	yes	Moderate
Singh	2011	no	yes	yes	no	yes	yes	no	yes	Moderate
Peng	2014	no	yes	yes	no	yes	yes	yes	yes	Moderate
Cheng	1995	yes	yes	yes	no	yes	yes	yes	yes	Low
Miskovic	2006	yes	yes	yes	yes	yes	yes	yes	yes	Low
Kan	2018	no	yes	yes	no	yes	yes	yes	yes	Moderate
Rajesh	2000	no	yes	yes	no	yes	yes	yes	yes	Moderate
Miller	1993	no	yes	yes	no	yes	yes	no	yes	Moderate
Vaisbuch	2005	no	yes	yes	no	yes	yes	yes	yes	Moderate
Dolapcioglu	2009	no	yes	yes	yes	yes	yes	yes	yes	Low
Lee	2010	no	yes	yes	yes	yes	yes	yes	yes	Low
Moini, Ashraf	2011	no	yes	yes	no	yes	yes	yes	yes	Moderate
Buke	2013	no	yes	yes	yes	yes	yes	yes	yes	Low
Montes-de-Oca-Valero	1999	no	yes	yes	no	yes	yes	yes	no	Moderate
Piura	2008	no	yes	yes	yes	yes	yes	yes	yes	Low
Bovicelli	2004	no	yes	yes	no	yes	yes	yes	yes	Moderate
Wax	2003	no	yes	yes	no	yes	no	no	yes	High
Abbi	1999	no	yes	yes	no	yes	yes	no	yes	Moderate
Hamanoue	2005	no	yes	yes	no	yes	yes	no	yes	Moderate
Klatt	2006	no	yes	yes	yes	yes	yes	yes	yes	Low
Aguilera	2012	no	yes	yes	yes	yes	yes	yes	yes	Low
Johnson	2019	no	yes	yes	yes	yes	yes	yes	yes	Low
Raj	2019	yes	yes	yes	yes	yes	yes	yes	yes	Low
Aplay	2021	no	yes	yes	yes	yes	yes	yes	yes	Low
Lipi	2019	no	yes	yes	no	yes	yes	no	yes	Moderate
Albayrak	2010	no	yes	yes	yes	yes	yes	yes	yes	Low
Al Mouallem	2022	no	yes	yes	no	yes	yes	no	yes	Moderate
Jung	2023	no	yes	yes	no	yes	yes	no	yes	Moderate
McHenry	2021	no	yes	yes	no	yes	no	yes	yes	Moderate
Wang	2023	yes	yes	yes	yes	yes	yes	yes	yes	Low
Taira	2021	no	yes	yes	no	yes	no	yes	yes	Moderate
Yayna	2023	yes	yes	yes	yes	yes	yes	yes	yes	Low
Bursac	2023	yes	yes	yes	yes	yes	no	yes	yes	Low
Tsakiridis	2021	yes	yes	yes	no	yes	no	yes	yes	Low

Thompson	2022	no	yes	yes	yes	yes	yes	yes	yes	Low
Okumura	2014	no	yes	yes	No	yes	no	yes	yes	Moderate
Albers	2001	no	yes	yes	yes	yes	yes	yes	yes	Low
Rao	2015	no	yes	yes	yes	yes	yes	yes	yes	Low
Lambert-Messerlian	2005	no	yes	yes	no	yes	no	yes	yes	Moderate
Li	2024	no	yes	yes	yes	yes	yes	yes	yes	Low
Soriano-Estrella	2024	no	yes	yes	no	yes	yes	yes	yes	Moderate
Makary	2010	no	yes	yes	no	yes	yes	yes	yes	Moderate
Freis	2016	no	yes	yes	yes	yes	yes	yes	yes	Low
Shuja	2024	no	yes	yes	no	yes	yes	yes	yes	Moderate
Kawasaki	2016	no	yes	yes	yes	yes	yes	no	yes	Moderate
Zeng	2019	yes	yes	yes	yes	yes	yes	yes	yes	Low
Chu	2004	no	no	yes	yes	yes	no	no	yes	High
Rai	2014	yes	yes	yes	yes	yes	yes	yes	yes	Low
Rathod	2015	yes	yes	no	no	no	yes	yes	yes	Moderate
Tolcha	2022	yes	yes	yes	yes	yes	yes	no	yes	Low
Libretti	2023	yes	yes	yes	yes	yes	yes	yes	yes	Low
Rajasekaran	2021	no	yes	yes	yes	yes	yes	yes	yes	Low
Qu	2022	yes	yes	yes	yes	yes	yes	yes	yes	Low
Mora-Palazuelos	2023	yes	no	yes	yes	yes	yes	yes	yes	Low
Lin	2021	yes	yes	yes	yes	yes	yes	yes	yes	Low
Copeland	2010	no	no	no	no	yes	yes	no	yes	High
