# Supplementary material for: Hydatidiform Mole with Coexisting Normal Pregnancy: A Systematic Review and Individual Participant Data Meta-Analysis
Source: Medicina (Kaunas). 2025 Oct 1;61(10):1781. doi: 10.3390/medicina61101781 (PMC12566089; doi:10.3390/medicina61101781)

Supplementary material 2: Case report of hydatidiform mole coexisting with a live fetus

A 40-year-old pregnant woman, G3P1, was referred to our institution for a second-trimester ultrasound at 19+2 gestational weeks. She conceived spontaneously, and the first trimester was complicated by persistent vaginal bleeding. The anatomy scan revealed a single fetus with no structural anomalies and a coexistent area with sonographic features suggesting a hydatidiform mole *(Figure 1).* A multiple conception with a normal co-twin with a complete or partial mole was suspected. Serum b-HCG concentration at 19+2 weeks of gestation was 581,900 mIU/mL, and amniocentesis results showed a 46 XY karyotype. The MRI was performed to rule out a molar infiltration of the myometrium, and it showed a normal anterior placenta and a concurrent area with cystic spaces ("Swiss cheese pattern") on the posterior uterine surface, which was not infiltrating the myometrium *(Figure 2).* The couple decided to continue the pregnancy after counseling regarding the obstetric and oncological risks associated with multiple conceptions and a concomitant hydatidiform mole.

Serum b-HCG concentrations were followed up, and a drop from 581,900 mIU/mL at 19+2 weeks to 39,316 mIU/mL at 34 weeks of gestation was reported. The cystic mass size was also reduced from 12x12x6cm at 19 weeks to 8.5x3.2 cm at 33 gestational weeks *(Figure 3).* The pregnancy was complicated by hyperthyroidism, which was effectively managed with the administration of propylthiouracil.

The patient was admitted to our department for labor onset at 36+1 weeks of gestation. A thorough counseling regarding the delivery mode was performed, and the patient opted for a vaginal delivery. The woman delivered vaginally an appropriate for gestational age neonate (birthweight of 2700 grams) with an Apgar score of 10-10. The third stage of labor was uncomplicated and characterized by a spontaneous delivery of the placenta and a separated mass of vesicular tissue *(Figure 4).* Total blood loss was 300mL. Subsequently, the woman underwent uterine suction and curettage to ensure the complete removal of the molar tissue. The placenta and the molar pregnancy were sent for histological examination *(Figure 5).* The molar specimen showed a triploid karyotype, confirming the diagnosis of partial molar pregnancy. The puerperium was uneventful. Serum b-HCG concentration was 3,652 mIU/ml the day after delivery, and it dropped to 10 mIU/ml two weeks after patient discharge. A negative b-HCG result was obtained at the five-month follow-up. Neonatal and pediatric outcomes were good, and the child was healthy, with normal acquisition of developmental milestones at the age of two. The patient provided informed consent to publish this case.

Figure legend

Figure 1-A transabdominal ultrasound image showing a mass attached to the posterior surface of the uterine cavity of 14.26 x 4.7cm with enlarged cystic spaces ("Swiss cheese").

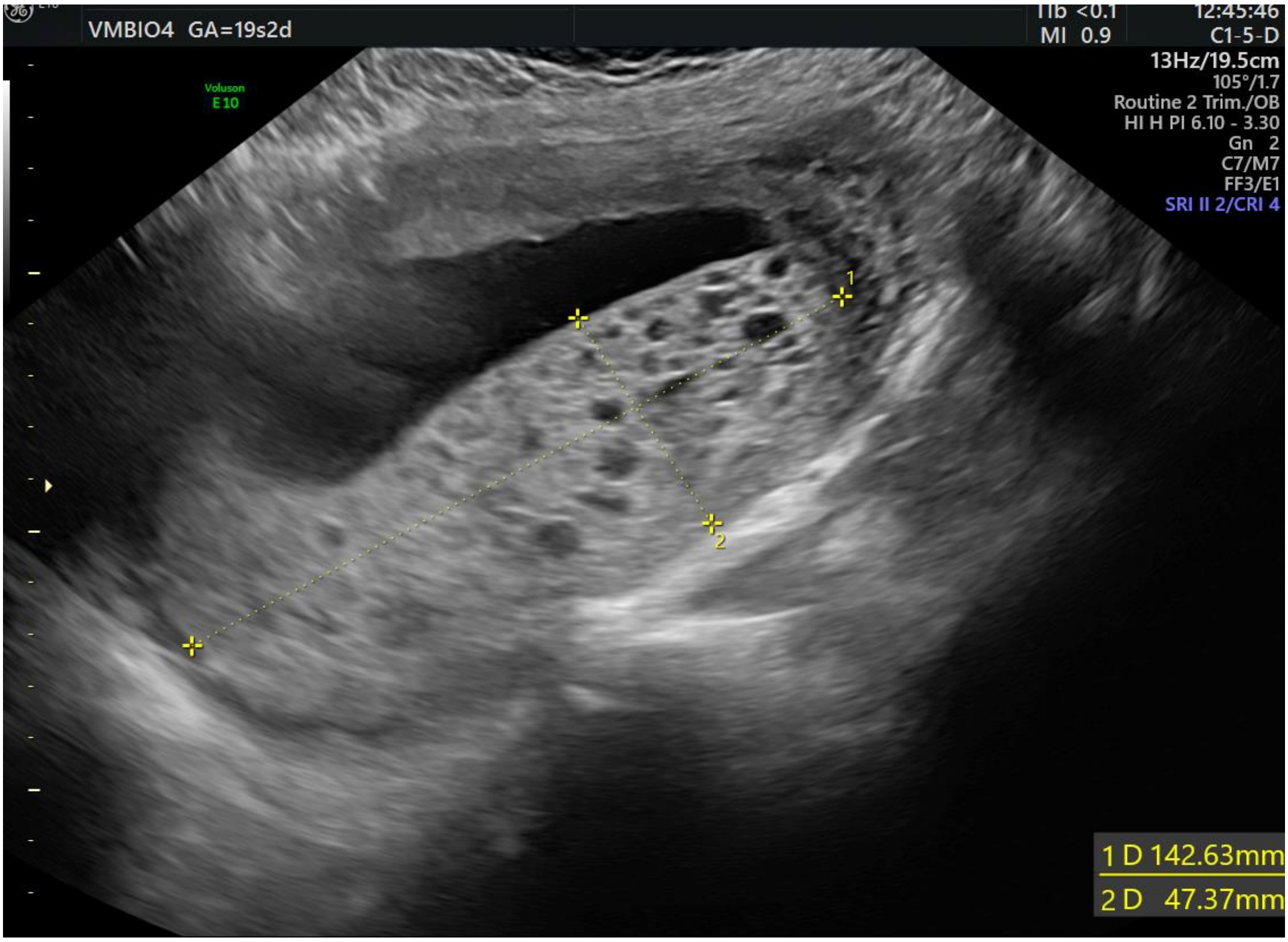

Figure 2- Fetal MRI imaging at 20+6 weeks' gestation. A. The coronal view shows the fetus with cephalic presentation (*f) on the right and the molar pregnancy with a vacuolated aspect (*m) on the left. B. The sagittal view displays the fetus (*f) and a regularly inserted placenta (*p) on the anterior surface of the uterine cavity. The hydatidiform mole (*m) is attached to the posterior surface and it does not infiltrate the myometrium. C. The transverse view displays the relationship between the anterior placenta (*p) and the posterior molar pregnancy (*m). The fetus is labelled with *f.

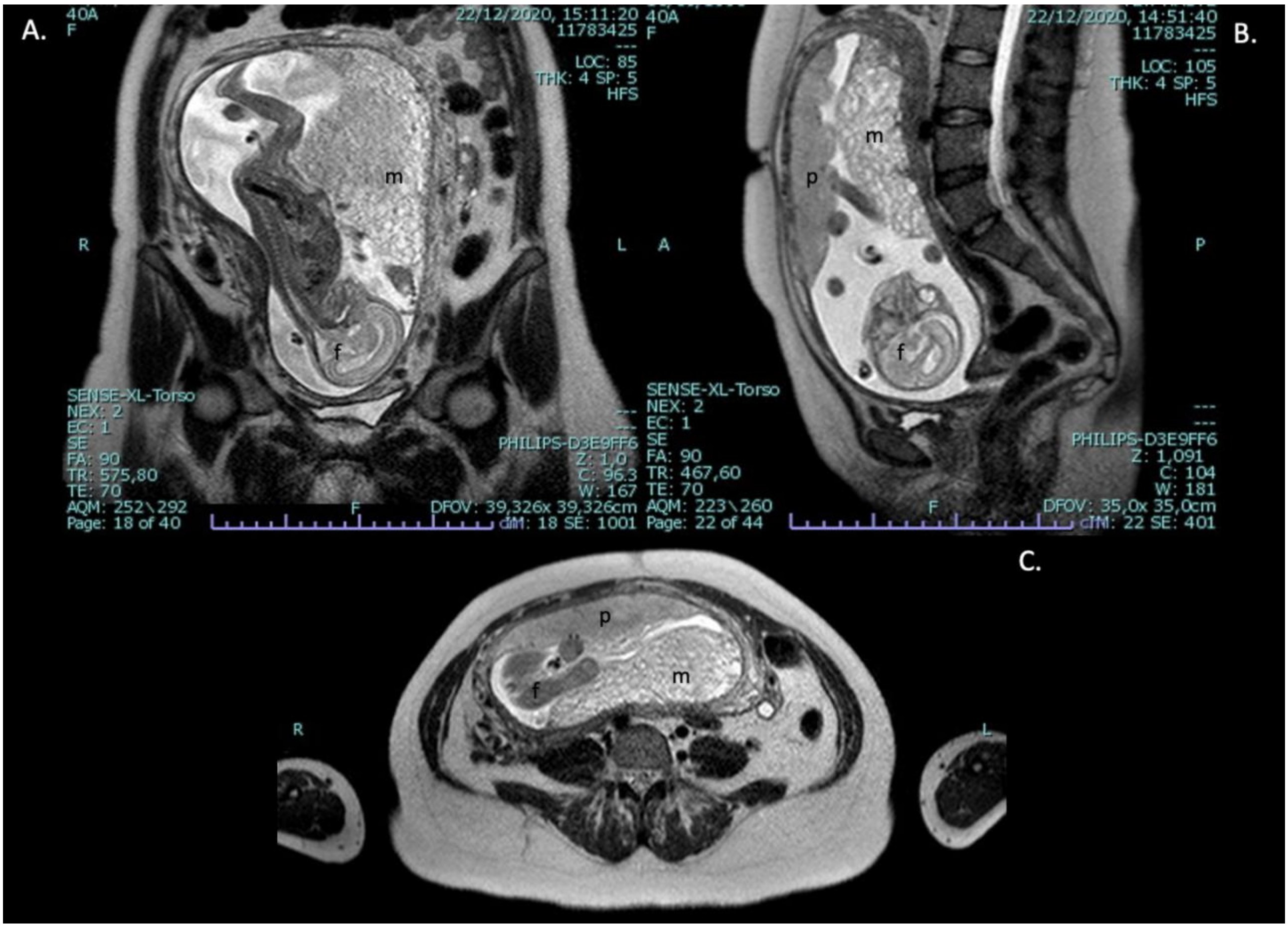

Figure 3 - The sequence of ultrasound images reporting a progressive involution (from A to C) of the molar mass size. A. 14.26cm (19+2 weeks'); B. 11.38cm (21+6 weeks'); C. 10.84cm (29+2 weeks').

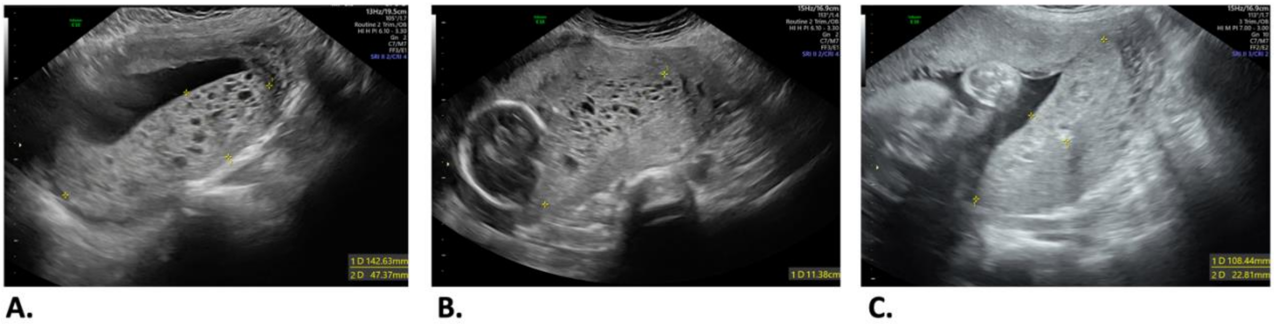

Figure 4 - On the right, the complete delivered placenta (*p) with size (17x16x3cm) and weight (638g) > 95th centile for gestational age at delivery. The amnio-chorial membranes are lifted to be displayed (*m) and the umbilical cord (*c) is clamped. On the left, the molar mass (green arrow) with a vacuolated aspect was delivered separately after the placenta.

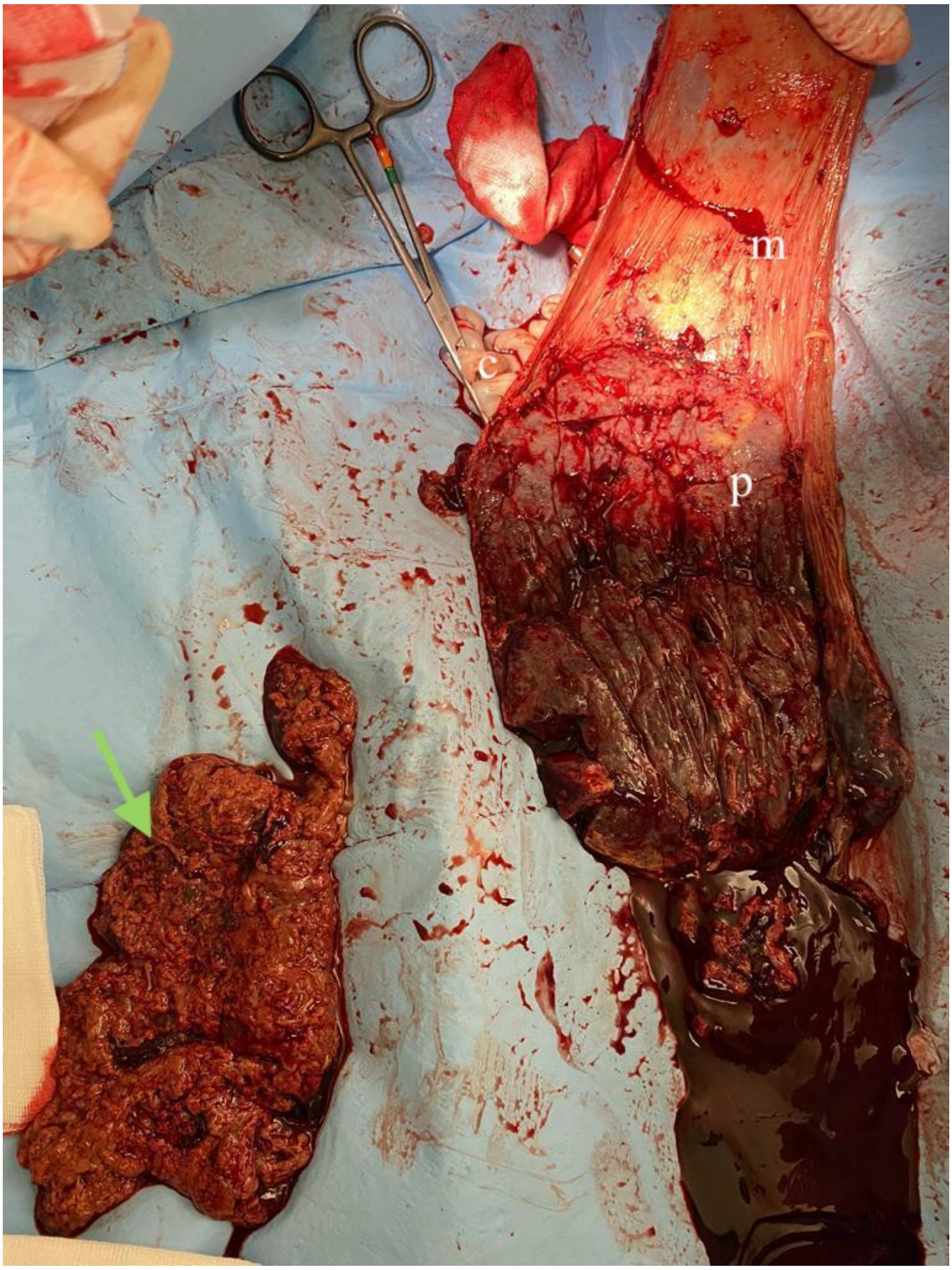

Figure 5 - Histopathological images showing A. the term placenta with increased syncytial knots (H&E 2.5x); B. the trophoblastic proliferation and atypia (H&E 40X); C. a shadow of necrotic hydropic villi (H&E 5x).

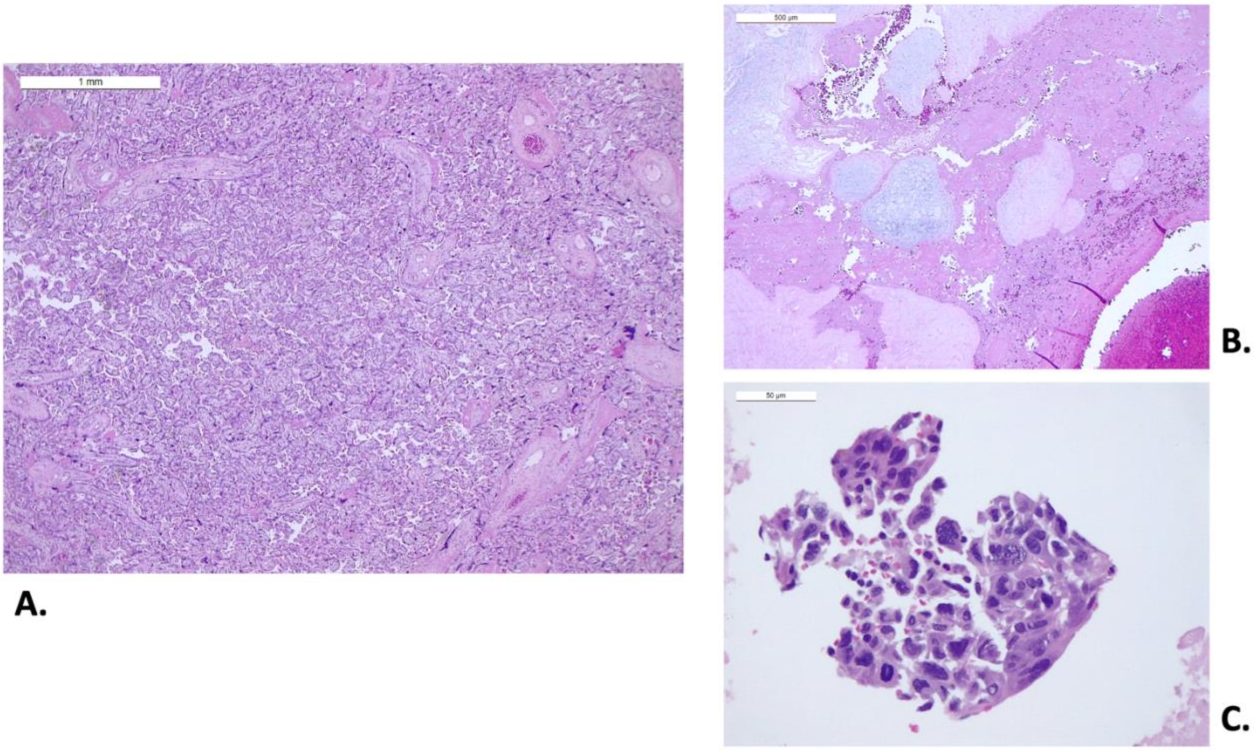

Supplement: Supplementary file 1 [file medicina-61-01781-s001.zip › supp-medicina-3822615/Supplementary Materials File S2.pdf]
