# Supplementary material for: Hydatidiform Mole with Coexisting Normal Pregnancy: A Systematic Review and Individual Participant Data Meta-Analysis
Source: Medicina (Kaunas). 2025 Oct 1;61(10):1781. doi: 10.3390/medicina61101781 (PMC12566089; doi:10.3390/medicina61101781)
Supplement: Supplementary file 1 [file medicina-61-01781-s001.zip › supp-medicina-3822615/Supplementary Materials File S4.pdf]

| Supplementary Material 4: Reported cases and their risk of bias according to the Joanna Briggs Institute (JBI) Critical Appraisal Checklist for Case Series | | | | | | | | | | | | |
|---|---|---|---|---|---|---|---|---|---|---|---|---|
| **Author** | **Year** | **Were there clear criteria for inclusion in the case series?** | **Was the condition measured in a standard, reliable way for all participants included in the case series?** | **Were valid methods used for identification of the condition for all participants included in the case series?** | **Did the case series have consecutive inclusion of participants?** | **Did the case series have complete inclusion of participants?** | **Was there clear reporting of the demographics of the participants in the study?** | **Was there clear reporting of clinical information of the participants?** | **Were the outcomes or follow up results of cases clearly reported?** | **Was there clear reporting of the presenting site(s)/clinic(s) demographic information?** | **Was statistical analysis appropriate?** | **Risk of bias** |
| Fishman | 1998 | yes | yes | yes | yes | yes | no | no | yes | no | no | Moderate |
| Niemann | 2007 | yes | yes | yes | yes | yes | yes | yes | no | yes | no | Low |
| Massardier | 2009 | yes | yes | yes | yes | yes | no | yes | yes | yes | no | Moderate |
| Kutuk | 2014 | yes | yes | yes | yes | yes | no | no | no | yes | no | Moderate |
| Giorgione | 2017 | yes | yes | yes | yes | yes | no | no | no | yes | no | Moderate |
| Lin | 2017 | yes | yes | yes | yes | yes | no | no | no | no | yes | Moderate |
| Liang | 2022 | yes | yes | yes | yes | yes | no | yes | yes | yes | yes | Low |
| Lu | 2022 | yes | yes | yes | yes | yes | yes | no | no | yes | no | Moderate |
