# Supplementary figures and images for: Hydatidiform Mole with Coexisting Normal Pregnancy: A Systematic Review and Individual Participant Data Meta-Analysis
Source: Medicina (Kaunas). 2025 Oct 1;61(10):1781. doi: 10.3390/medicina61101781 (PMC12566089; doi:10.3390/medicina61101781)

## Supplementary Material 5: Bar chart of risk of bias assessment

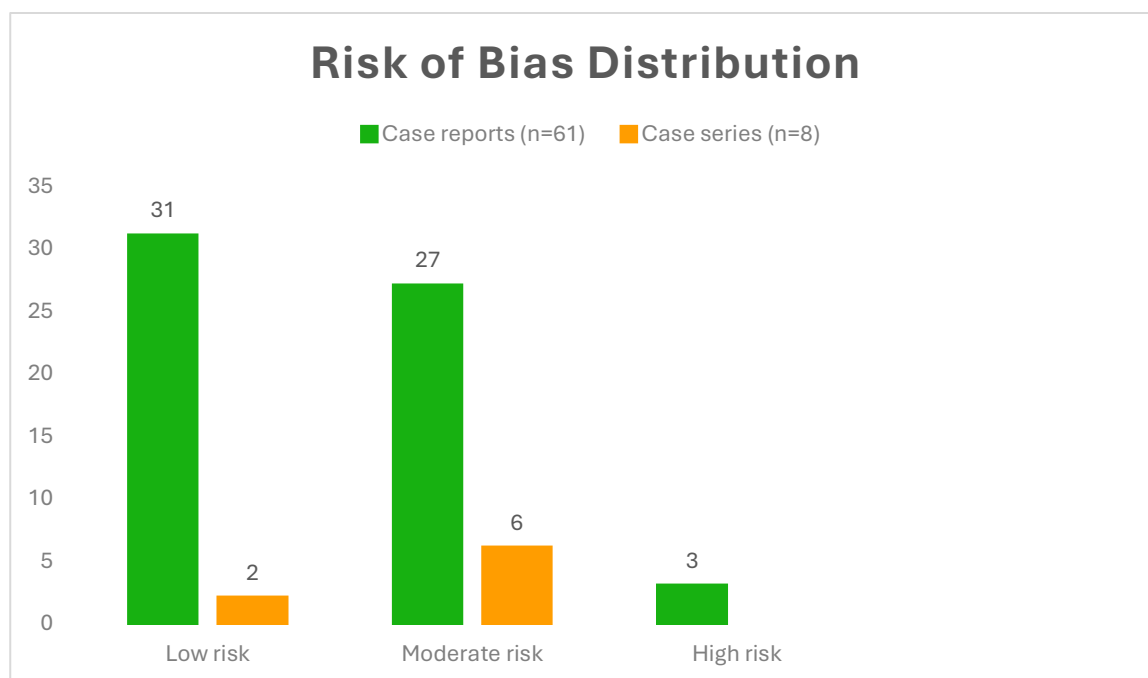

Supplement: Supplementary file 1 [file medicina-61-01781-s001.zip › supp-medicina-3822615/Supplementary Materials File S5.pdf]
